# The complete chloroplast genome sequence of *Sorbus amabilis* (Rosaceae)

**DOI:** 10.1080/23802359.2020.1768951

**Published:** 2020-05-27

**Authors:** Xingwang Zhang, Yanping Xie, Shuping Xiao, Xiaomin Wu

**Affiliations:** a School of Life Sciences, Huaibei Normal University, Huaibei, China;; b School of Information, Huaibei Normal University, Huaibei, China;; c Forestry Bureau of Mingxi County, Sanming, China

**Keywords:** Chloroplast genome, *Sorbus amabilis*, phylogenetic analysis

## Abstract

*Sorbus amabilis* Cheng ex Yü, a small excellent ornamental tree species, is only distributed in Eastern China. In this study, we assembled and annotated the complete chloroplast (cp) genome of the species using the next-generation sequencing for the first time. The cp genome was 160,006 bp in size, consisting of two copies of invert repeat (IR) regions of 26,405 bp, one large single-copy (LSC) region of 87,870bp, and one small single-copy (SSC) region of 19,326 bp. The overall GC content of the genome was 36.55%. The genome was predicted to contain 128 genes, including 88 protein-coding genes, 37 tRNA genes, and eight rRNA genes. Phylogenetic analysis of 25 chloroplast genomes in Rosaceae indicated that *S*. *amabilis* is most closely related to *S*. *commixta*. These findings may provide useful information to the phylogeny of the genus *Sorbus*.

*Sorbus amabilis* Cheng ex Yü is a small deciduous broad-leaved tree species belongs to the genus *Sorbus* of the family Rosaceae. It can only be found in eastern China and grows at an altitude ranging from 900 to 2100 m above sea level. As an excellent ornamental plant, it is very beautiful with pinnate leaves, white flowers, and red fruits. Furthermore, *S. amabilis* is also considered as an important economic tree species for producing timber and extracting medicine (Zhang et al. 2018). The taxonomy of *Sorbus* and its subgenus group classification is very complicated due to the existence of polyploidy gametophytic apomixis and natural hybridization within genus (Wang et al. 2020). The chloroplast (cp) genome has been extensively applied in species identification, uncovering plant phylogeny and evolution and in recent years, therefore the complete cp genome of *S. amabilis* was firstly determined based on the next-generation sequencing technology, which could provide more informatics data for the phylogeny of *Sorbus*.

Fresh leaves of *S. amabilis* were sampled from Shixin peak, Huangshan Mountain, Anhui Province, China (30.14°N, 118.18°E). The voucher specimens were preserved at the Key Laboratory of Plant Resource and Biology in Huaibei Normal University with the accession number of HS20190526007. Total DNA extraction and whole genome sequencing were conducted by Nanjing Genepioneer Biotechnologies Inc. (Nanjing, China) with the Illumina Hiseq X Ten platform. Raw data was filtered using the fatap 0.20.0 (Chen et al. 2018). A total of 18,903,785 clean reads were produced and assembled by NOVOplasty 2.7.2 (Dierckxsens et al. 2016). Annotation was performed using the CpGAVAS pipeline (Liu et al. 2012) and BLAST searches and coupled with manual correction to remove errors and redundant annotations.

The complete cp genome of *S. amabilis* (GenBank accession number MT357029) was 160,006 bp in length and exhibited a typical quadripartite structure, which was composed of two copies of invert repeat (IR, 26,405 bp) regions separated by a large single-copy (LSC, 87,870 bp) and a small single-copy (SSC, 19,326 bp) regions. The overall GC content was 36.55%, while the corresponding values of LSC, SSC, and IR regions were 33.59%, 30.24%, and 42.63%, respectively. A total of 128 genes were annotated in the cp genome, including 88 protein-coding genes, 37 tRNA genes, and eight rRNA genes. Seven protein-coding genes, eight tRNA genes, and four rRNA genes were duplicated in the IR regions. Moreover, we also found that 17 genes contain introns, 14 of which contained one introns, the other 3 of genes (*clpP*, *rps12*, and *ycf3*) contain two introns.

To investigate phylogenetic position of *S*. *amabilis*, the sequence alignment was performed on the 25 cp genome sequences using MAFFT 7.307 (Kazutaka and Standley 2013), including 23 Maloideae species and two Prunnoideae species as outgroup. The maximum likelihood (ML) tree was constructed by online RAxML BlackBox software based on rapid bootstrap algorithm (Stamatakis et al. 2008). Our results indicated that *S. amabilis* is most related to *S. commixta* with very high bootstrap support values, and formed a clade sister with *Pyrus*, and the other *Sorbus* species are close to *Malus* species (Figure 1). The current phylogenetic relationship was consistent with the results of Niu et al. (2019) based on cp genome sequences.

**Figure 1.**
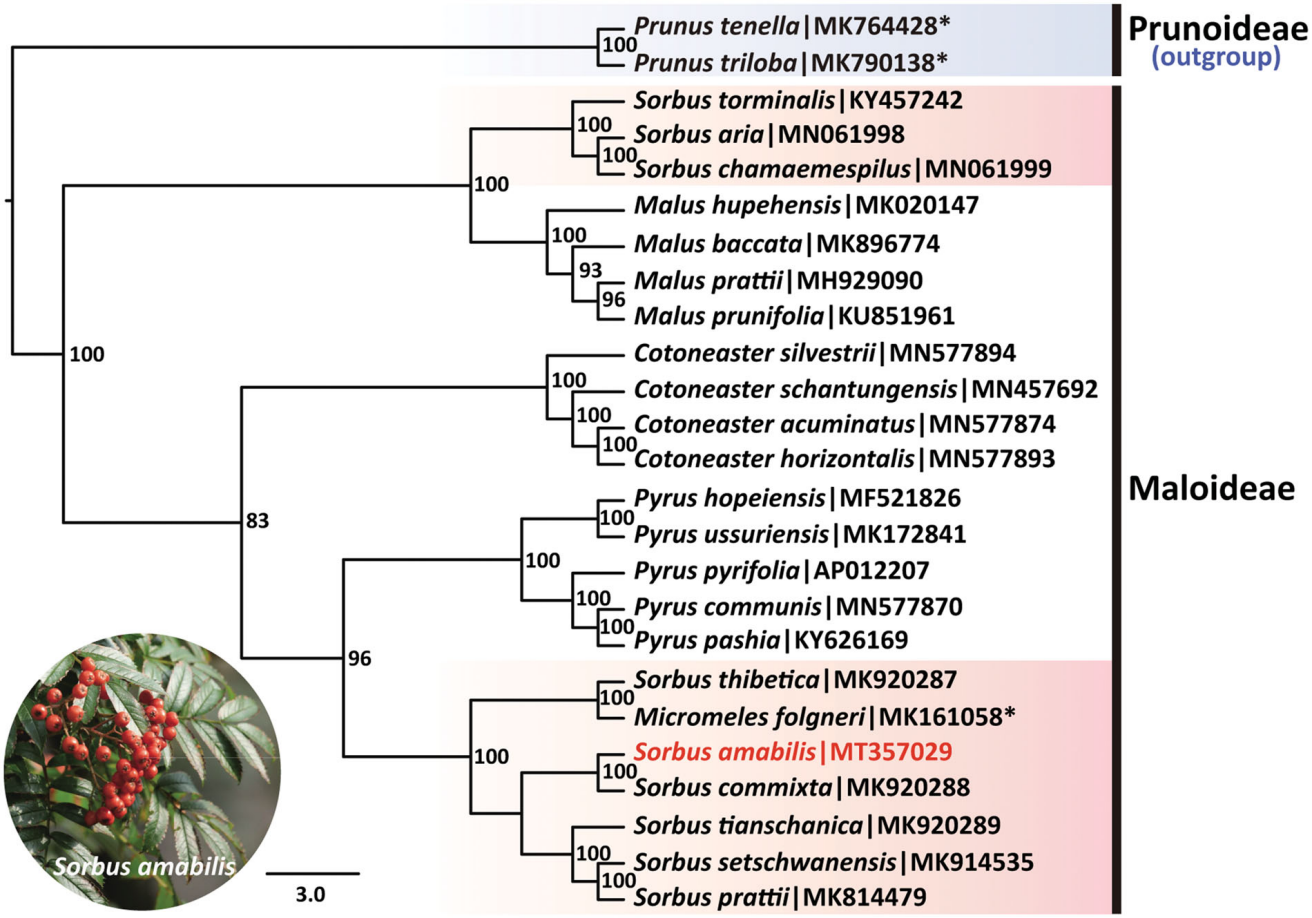
Phylogenetic tree inferred by maximum likelihood (ML) method based on cp genome sequences of 25 Rosaceae species. The bootstrap support values are labeled for each node based on 1000 replicates. The species names marked with asterisk symbol were *Prunus triloba*, *Prunus tenella*, and *Micromeles folgneri* in NCBI, which were the synonym of *Amygdalus triloba*, *Amygdalus nana*, and *Sorbus folgneri* in Flora of China, respectively.

## Data Availability

The complete chloroplast genome sequence and annotation of *Sorbus amabilis* that support the findings of this study are openly available in Zenodo at https://doi.org/10.5281/zenodo.3777235.
